# Parenthood and Well-Being: The Moderating Role of Leisure and Paid Work

**DOI:** 10.1007/s10680-016-9391-3

**Published:** 2016-08-23

**Authors:** Anne Roeters, Jornt J. Mandemakers, Marieke Voorpostel

**Affiliations:** 1 Utrecht University, Utrecht, The Netherlands; 2 Netherlands Institute of Social Research, The Hague, The Netherlands; 3 Wageningen University, Wageningen, The Netherlands; 4 Swiss Centre of Expertise in the Social Sciences (FORS), Lausanne, Switzerland

**Keywords:** Fixed effects models, Gender, Lifestyles, Heterogeneity, Transition to parenthood, Well-being

## Abstract

This study contributes to our knowledge on the association between parenthood and psychological well-being by examining whether pre-parenthood lifestyles (leisure and paid work) moderate the transition to parenthood. We expected that people with less active lifestyles would find it easier to adapt to the demands of parenthood. Using eleven waves of the Swiss Household Panel (*N* = 1332 men and 1272 women; 1999–2008, 2010), fixed effects models are estimated for men and women separately. Results show that—on average—parenthood was not associated with well-being for men, whereas it increased well-being for women. As expected, the well-being premium/cost to parenthood was contingent upon individuals’ lifestyle before the transition to parenthood. For men, parenthood reduced well-being, but only if they frequently participated in leisure before the birth of the child. For women, motherhood had a beneficial effect on well-being but this effect was weaker for women who combined leisure with working long hours before motherhood.

## Introduction

The transition to parenthood has gained a lot of attention in recent societal and scholarly debates. Although the literature showed that children bring rewards as well as costs, a number of scholars argue that parenthood is harmful to well-being because children inhibit (couple) leisure and create role overload and conflict (Claxton and Perry Jenkins 2008; Nelson et al. 2014; Nomaguchi and Milkie 2003; Treas et al. 2011; Umberson et al. 2010). Yet, whereas these studies assumed that all individuals who make the transition to parenthood encounter similar problems combining work, leisure, and parenthood, we contend that individuals who seldom participated in leisure before the transition to parenthood do not need to adapt their lifestyles as much as their more active counterparts. Therefore, variations in individuals’ leisure before the transition to parenthood may explain part of the heterogeneity in the effects of parenthood on well-being.

Well-being here is measured as the absence of negative emotional states such as depression. We conceptualize lifestyles as individuals’ participation in leisure activities and paid work. With the term leisure, we refer to individuals’ participation in activities, such as sports, socializing, and cultural participation. We believe that by looking at these “truly elective uses of time” (Mattingly and Bianchi 2003: 1010), we capture individuals’ lifestyles best. We also pay attention to paid work hours, because it is a central lifestyle characteristic that affects the possibilities for combining parenthood and leisure. Another reason why it is interesting and relevant to study both leisure and work hours is that the two substitute each other to a great extent (Gershuny 2000). We are mostly interested in the limits children impose upon leisure, as the intrinsic value of leisure is higher (Kahneman and Krueger 2006), and we expect the costs of forgoing leisure are larger than the costs of reducing paid work.

The analyses are based on eleven waves of the Swiss Household Panel (SHP, *N* = 1332 men and 1272 women, 1999–2008, 2010). Switzerland is an interesting context to study the link between well-being and the transition to parenthood, because welfare state support for parents is limited and work–family reconciliation policies are scarce (Bertozzi and Gilardi 2008). In such a context, the role of individual preferences, resources, and restrictions may be especially pronounced. The SHP is a useful data-set because it contains yearly data on well-being and a wide range of leisure activities. We estimate fixed effects models to investigate the impact of parenthood, net of time-invariant individual characteristics such as personality traits. Because the division of labor and nature of leisure is strongly gendered (Bianchi and Milkie 2010), men and women are analyzed separately.

This study answers the following research question: To what extent is the influence of parenthood on well-being moderated by individuals’ participation in leisure and paid work? The study’s main contribution is that it adds to existing knowledge on the conditions under which parenthood is more or less beneficial for individual well-being (Barban 2013; Keizer and Schenk 2012; Myrskylä and Margolis 2014; Nomaguchi 2012), by differentiating between parents with varying lifestyles. Although the potential relevance of parents’ lifestyle is often mentioned in the literature, few studies actually look into this. Some studies controlled for parents’ activities before the transition to parenthood (e.g., Knoester and Eggebeen 2006; Nomaguchi and Milkie 2003), or studied the possibility that changes in paid work and leisure mediate the effect of parenthood on well-being (Claxton and Perry Jenkins 2008; Keizer and Schenk 2012; Pollmann-Schult 2014). No study we are aware of explicitly investigated the *moderating* role of lifestyles and considered the possibility that pre-parenthood lifestyle (in terms of leisure and paid work) changes how parenthood affects well-being. Furthermore, our study adds to the literature on the consequences of role overload and scaling back (e.g., Bianchi and Milkie 2010; Treas et al. 2011), by investigating the role of participation in leisure and paid work during the transition to parenthood.

## Background and Hypotheses

### Parenthood and Well-Being: A Rewards and Costs Approach

Research on the impact of parenthood on well-being is abundant and characterized by a wide variety in theories, measures, samples, and methods. There is no scholarly consensus on the net effect of parenthood: Whereas some studies found that parenthood increases well-being (e.g., Kohler et al. 2005; Myrskylä and Margolis 2014), others found that it has a negative effect (e.g., Evenson and Simon 2005). There is an emerging consensus that “[p]arenthood, *per se*, does not predict well-being in a systematic way” (Umberson et al. 2010: 614). The impact of parenthood on well-being is contingent upon factors such as parental education (Myrskylä and Margolis 2014) and the age and number of children (Kohler et al. 2005; Nomaguchi 2012; see also Nelson et al. 2014; Umberson et al. 2010, for extensive reviews of the literature.) Below we provide a concise overview of ideas and evidence on the rewards and costs of parenthood.

#### Rewards

Children induce positive emotions (such as love and affection), a sense of meaning or purpose, and an experience of psychological growth (Liefbroer 2005; Morgan and King 2001; Nelson et al. 2014; Nomaguchi and Milkie 2003; Umberson and Gove 1989; Woo and Kelly Raley 2005). It has also been argued that parenthood is associated with higher levels of social integration. (New) parents have more frequent contact with relatives, friends, and neighbors (Knoester and Eggebeen 2006; Nomaguchi and Milkie 2003), provide more care to kin and non-kin outside the household (Gallagher and Gerstel 2001), and are more involved in service-oriented activities (Knoester and Eggebeen 2006).

#### Costs

The literature on the costs of parenthood is more abundant than the literature on its rewards (Nomaguchi and Milkie 2003). Often, scholars argue that the impact of parenthood is indirect, because it increases certain stressors that, in turn, decrease well-being. Examples of stressors are financial distress and marital conflict. Because children are expensive, parents experience more financial struggles compared to their childless counterparts (Bird 1997; Nelson et al. 2014). Children also impose pressure on the partner relationship: Partners have to renegotiate the division of labor, spend less quality time together, and experience a decrease in the level of sexual intimacy (Kluwer and Johnson 2007; Nelson et al. 2014; Nomaguchi and Milkie 2003; Twenge et al. 2003; Umberson et al. 2010).

Consistent with the arguments on the rewards and costs of children, we expect that the transition to parenthood may be associated with either a decrease or increase in well-being. We label these base expectations Hypothesis 1a (predicting a decrease) and 1b (predicting an increase). Because prior research showed that the impact on individuals’ lives is strongest when children are young (Ishii-Kuntz and Seccombe 1989; Munch et al. 1997; Myrskylä and Margolis 2014; Nelson et al. 2014; Umberson et al. 2010), we also consider the amount of time since the birth of the child when testing the association between parenthood and well-being.

### Prior Research on the Role of Leisure and Paid Work

Most studies on the transition to parenthood consider individual’s time spent in leisure and paid work as an important aspect because children curtail these activities and create role overload. Nevertheless, few studies on the transition to parenthood and well-being specifically tested the role of leisure and paid work. The studies that did mostly looked at paid work hours and start from the idea that children reduce well-being because parents have to balance multiple demands, which creates role overload and conflict.

The empirical results are inconclusive. For example, Wethington and Kessler (1989) found that women’s changes in the work domain (e.g., dropping out of employment) affected changes in psychological distress, net of the impact of parenthood. Keizer et al. (2010) found that relatively large reductions *and* increases in women’s (but not men’s) work involvement following parenthood reduced mental health. Keizer and Schenk (2012) find almost no effects of changes in paid and unpaid work on marital satisfaction.

The literature on the role of changes in leisure is more limited. Although several studies propose that one of the costs of children is that they curtail leisure (Claxton and Perry Jenkins 2008; Goldberg and Perry Jenkins 2004; Nelson et al. 2014; Twenge et al. 2003; Wethington and Kessler 1989), few studies investigate the role of changes in leisure during the transition to parenthood. Children require direct care and supervision and increase the amount of cleaning, laundry, and cooking that has to be done (Craig and Bittman 2008; Gjerdingen and Center 2005; Nomaguchi and Milkie 2003; Sayer 2005). As a result, child care demands compete with leisure, (paid) work, and sleep (Claxton and Perry Jenkins 2008; Nomaguchi and Milkie 2003; Umberson et al. 2010). Children also decrease parents’ flexibility to do whatever they want, whenever they want (Liefbroer 2005; Twenge et al. 2003; Umberson and Gove 1989). Because leisure has a high intrinsic value and is associated with high levels of positive affect (Kahneman and Krueger 2006; Nomaguchi and Milkie 2003; Umberson et al. 2010), the decrease in leisure (flexibility) is likely to harm well-being. Moreover, when less time is spent in leisure, relationships with partner and friends may decrease in quality, and there are fewer opportunities to recharge (Claxton and Perry Jenkins 2008; Dew and Wilcox 2011; Nelson et al. 2014; Shaw 2008; Twenge et al. 2003).

Finally, prior research showed considerable gender differences with regard to paid work and leisure. Women and men vary in their leisure time and activities, have different preferences in leisure, and experience leisure differently (e.g., Bittman and Wajcman 2000; Henderson and Hickerson 2007; Mattingly and Bianchi 2003).

### The Moderating Role of Leisure and Paid Work

In this section, we go beyond the studies that focus on changes in leisure and paid work after the birth of the first child by formulating expectations on the role of pre-parenthood leisure and paid work. Our main expectation is that the impact of parenthood is contingent upon parents’ involvement in leisure before the birth of the child. Most transition to parenthood studies implicitly or explicitly assumed that childless individuals spend a substantial proportion of their time in leisure. In reality, individuals’ lifestyles vary. Whereas some people frequently participate in active leisure, others may spend more time in paid work, non-leisure (e.g., domestic labor), or more passive forms of leisure (e.g., watching television).

Those with relatively active lifestyles can respond to the birth of a child in two ways: (1) by scaling back and curtailing leisure or (2) by maintaining their old lifestyle as much as possible. In the first case, we expect that the well-being of the new parent decreases. By reducing the frequency of leisure activities, they lose the associated benefits, such as close friendships and the health-effects of exercising. This is likely to be particularly harmful for “active” parents, because their earlier involvement suggests that they highly value these activities. In the second case, in which the new parent maximizes his or her leisure, we may also see a decrease in well-being. The role strain approach predicts that parents who accumulate demands are more likely to experience role overload and role conflict (e.g., Bianchi and Milkie 2010). For example, persons who are active in sports will experience difficulties combining this with (home oriented) child care. Multitasking and outsourcing may also create difficulties, because the former involves effort and distraction, whereas the latter may cause feelings of guilt. Thus, we expect that individuals who more often participated in leisure before the birth of their first child will experience a decrease in well-being after they become parents, regardless of their way to cope with this event.

The transition to parenthood is likely to have a different impact on individuals who participated less in leisure before the transition to parenthood. Because their leisure involvement before the birth of their child indicates that attach less meaning to an active lifestyle, they may not be affected if they have to scale back and reduce their leisure involvement. Moreover, if these parents decide not to scale back and retain their old (low) levels of leisure participation, they may be less likely to experience role overload because they are better able to accommodate children in their old schedule.

The arguments above lead us to expect that people who participated more in leisure before the birth of their first child experience a larger decrease in well-being following parenthood (Hypothesis 2a). Because empirical evidence on the effect of parenthood on well-being is inconclusive, we may find that the effect is positive. If this is the case, we expect that the strength of the *positive* effect is contingent upon individuals’ participation in leisure.

Long work hours also reflect an active lifestyle, so new parents who worked longer hours before parenthood may experience similar costs as those who had active leisure patterns. Thus, we hypothesize that people who participated more in paid work before the birth of their first child experience a larger decrease (or a smaller increase) in well-being following parenthood (Hypothesis 2b). Again, it may also be possible that the strength of a positive effect is contingent upon individuals’ pre-parenthood work hours.

We also investigate the possibility that the *combination* of paid work and leisure moderates the impact of parenthood on well-being. The demands of parenthood may be most difficult to accommodate for individuals who combined long hours with an active leisure pattern. These individuals are most likely to have a preference for an active lifestyle and to experience role overload if they maintain their pre-parenthood lifestyle. Therefore, people who participated more in both paid work *and* in leisure before the birth of their first child may experience a larger decrease (or a smaller increase) in well-being following parenthood (Hypothesis 3).

Hypothesis 2a, 2b, and 3 predict that parenthood is more harmful (or less beneficial) for parents who had active lifestyles before the birth of their child, regardless of their lifestyle after the child is born. We examine this presumption by investigating whether the main parenthood effect and the possible moderating effect of pre-parenthood lifestyles can be explained by taking individuals’ *time*-*varying* participation in leisure and paid work into account.

Our expectations are tested using fixed effect models. Men and women are analyzed separately. The analyses are based on Swiss data. Switzerland is a country characterized by limited welfare state support for parents and scarce work–family reconciliation policies (Bertozzi and Gilardi 2008), making women’s part-time work the main strategy to combine work and family (Giraud and Lucas 2009; Widmer and Ritschard 2009). Therefore, the role of individual preferences, resources, and restrictions may be especially pronounced and gender differences are likely to occur.

## Methods

### Data and Sample

For our study, we used the Swiss Household Panel (SHP). The SHP follows a random sample of households in Switzerland on an annual basis since 1999. Currently, there are fifteen waves available. Because wave 11 (2009), 13 (2011), 14 (2012), and 15 (2013) did not include information on all leisure activities, we only used the first ten waves (1999–2008), and wave 12 (2010). Data are collected using computer assisted telephone interviews (CATI). Every wave covers three types of questionnaires: (1) a household grid (used to assess the composition of the household); (2) a household survey; and (3) individual questionnaires. The individual questionnaires are targeted at all household members aged 14 or older.

The total sample is composed of two parts. The SHP started with a first sample in 1999 (SHP I) and was supplemented with a second sample in 2004 (SHP II). Both samples are representative of private households in Switzerland, stratified by region. The SHP I-sample consisted of 7799 respondents from 5074 households. The SHP II-sample was smaller with 3654 respondents from 2538 households. On the household level, response rates were 64 % in the first wave of the first sample (1999) and 65 % in the first wave of the second sample (2004). On the individual level, response rates (conditional upon household participation) were, respectively, 85 and 76 %. During the course of the panel, individuals entering the household were included as new sample members. Household members leaving the original household stayed in the sample and were included in the study as new households. The panel design was unbalanced, which means that respondents could temporarily exit the panel.

Attrition is a common issue in panel data (Groves 2006): In the SHP, attrition rates were relatively high in the first few waves, but overall non-response bias in the Swiss Household Panel is mild and comparable to other panel studies. Married couples are somewhat overrepresented and non-Swiss respondents, men, younger (14–24 years old), and lower educated respondents were more likely to drop out, as is the case in other large-scale household panels (Lipps 2007). About 65 % of the original 1999 and 2004 samples still participated in 2008.

We selected men and women aged 18–40 years old who did not have children at baseline (1827 men and 1723 women) and thus were still at risk of becoming parents. Respondents were followed from the moment they entered the sample. As a second restriction, we only included respondents for whom we have at least two waves of observation in order to be able to detect the transition to parenthood (1336 men, 1278 women and 14,000 observations in total). We applied list wise deletion of missing values, as there were relatively few missing values. The final sample size comprises 1332 men and 1272 women and a total of 13,944 observations. The SHP is a household panel, but we analyzed men and women separately, so the observations can be considered independent. A few cases of siblings of the same sex within the same household were observed, but additional tests showed that this did not affect the results.

### Measures

#### Well-Being

Well-being is measured as the absence of negative emotional states with a single item. The respondents were asked: “Do you often have negative feelings such as having the blues, being desperate, suffering from anxiety, or depression, if 0 means “never” and 10 “always?” We reverse-coded this measure so that higher values correspond with higher levels of well-being.

#### Transition to Parenthood

We constructed a dummy variable indicating whether individuals had a child at the interview date. This information was constructed based on the birth dates of reported children. We only took biological or adopted children into account. We also calculated the number of months since the transition to parenthood (i.e., birth of first child).

#### Involvement in Leisure

The interviewer asked the respondent the following question “I am now going to list a number of leisure activities. How frequently do you practice them?” Answer categories were (1 = *every day*, 2 = *at least once a week*, 3 = *at least once a month*, 4 = *less than once a month*, and 5 = *never*). Twelve activities returned in each questionnaire (only wave 12 differed slightly): (a) Meeting friends, acquaintances, or colleagues; (b) reading; (c) playing an instrument or singing; (d) Do it yourself or gardening; (e) attending courses (except vocational training) (not asked in wave 12 and therefore excluded); (f) going to a disco, a dance hall, or a techno party; (g) going to a bar, pub, or restaurant; (h) practicing an individual or team sport; (i) walking (includes also hiking in wave 12); (j) attending sports events; (k) going to the theater, the opera, visiting an exhibition (split in three separate questions in wave 12); (l) going to the cinema. These activities reflect the most popular leisure activities in Switzerland (SFSO 2005). We exclude television watching because this does not bring the same benefits as other leisure activities do (Craig and Mullan 2013). A scale was constructed by taking the average over the eleven items [all items except item (e)]. The original answer categories were recoded so that higher values corresponded with a higher frequency (0 = *never* through 4 = *every day*).

In order to capture the role of leisure before parenthood, we constructed a time-invariant variable that measures the average involvement over the waves during which the respondent is childless. Thus, for those respondents who make the transition to parenthood, the time-invariant variables measure the average involvement before the transition to parenthood (i.e., average leisure frequency). By interacting this time-invariant variable with the transition to parenthood dummy, we can analyze whether the impact of parenthood is contingent upon pre-parenthood leisure for those who become parents. In addition to this time-invariant variable, we also constructed a time-varying variable, measuring leisure involvement in each wave. This measure enables us to investigate whether changes in leisure are associated with changes in well-being.

#### Involvement in Paid Work

Employed respondents were asked the question “How many hours per week do you usually work each week for your main job?” Respondents were instructed to include usual paid and unpaid overtime. Non-employed respondents were assigned a value of 0 on the work hours variable. We used the information on the respondents’ work hours to construct variables similar to those for leisure. We control for employment status (0 = *non-employed*, 1 = *employed*) in models that include the time-variant work hours variable to better capture changes in work.

#### Control Variables

Because we use fixed effects models, time-invariant characteristics are canceled out and only time-varying characteristics remain. The control variables are age of the respondent and respondents’ civil status (single, cohabiting but unmarried, married) and self-reported general health (5 point scale, higher values indicate a better health). These variables are well-known correlates of well-being and are also determinants of work and leisure involvement. For example, prior research in Switzerland showed that younger individuals spend a larger share of their leisure time outside of their home, and spend more time with friends and making music than older persons, who are more active in gardening and hiking (SFSO 2005). Because one would expect that women and their partners adjust their lifestyle during pregnancy and many studies reported a child anticipation effect on well-being (Myrskylä and Margolis 2014), we also constructed a dummy variable, indicating that the interview took place within 6 months before the birth of the first child. This variable was labeled as “pregnancy of partner” (for men)/“pregnancy” (for women).

### Analytical Method

We employ fixed effect models to analyze changes in well-being following parenthood. Fixed effects models have the advantage that all observed and unobserved) time-invariant individual characteristics (e.g., personality, work-leisure preferences) that may bias the parenthood effect are canceled out. We ran the analyses separately for men and women.

The analysis proceeds in five steps. First, the baseline model estimates the average relationship between well-being and becoming a parent (Model 1). This analysis replicates previous findings on the transition to parenthood and tests Hypotheses 1a and 1b. Second, in Model 2, we test Hypothesis 2a by investigating whether the average association between parenthood and well-being obscures differences between parents who had active lifestyles before parenthood versus those who were less active. We do so by including an interaction term between the time-invariant pre-parenthood leisure indicator and the transition to parenthood dummy variable. Note that the main effect of the interaction cannot be estimated because pre-parenthood leisure is a time-constant covariate. In the third and fourth step, we investigate the role of paid work by including interactions between the transition to parenthood dummy and pre-parenthood work hours (Model 3) as well as a three-way interaction between parenthood, pre-parenthood leisure, and pre-parenthood work hours (Model 4). These models test, respectively, Hypothesis 2b and Hypothesis 3. Fifth, the final model (Model 5) includes time-varying indicators of leisure and work. This additional model tests whether changes in leisure and work account for the moderating effect of pre-parenthood lifestyles.

## Results

### Descriptive Analyses

Tables 1 and 2 show descriptive statistics for men and women, respectively. Three groups of observations are distinguished: (1) observations of individuals who do not make the transition to parenthood during the period of observation, (2) observations of (future) parents before the transition to parenthood, and (3) observations of parents after the transition to parenthood.

**Table 1:** Well-being, parenthood variables, lifestyle variables, and parental background variables: descriptive statistics for men

Variable (range)	No transition to parenthood		Pre-parenthood		After parenthood	
	Mean	SD	Mean	SD	Mean	SD
Well-being (0–10)	8.22	1.86	8.66^a^	1.58	8.53^a^	1.70
Optimism (0–10)	7.36	1.71	7.64^a^	1.59	7.51^a^	1.46
Life satisfaction (0–10)	7.80	1.37	8.17^a^	1.19	8.10^a^	1.16
Satisfaction with free time (0–10)	6.58	2.36	6.30	2.39	5.88	2.38
Satisfaction with leisure act (0–10)	7.68^a^	1.89	7.77^a^	1.78	7.17	2.04
Leisure (0.27–3.27)	1.93	.39	1.98	.35	1.84	.36
Pre-parenthood leisure (1–2.73)	–	–	1.98^a^	.28	1.97^a^	.28
Work hours (0–90)	35.80	17.38	41.49	12.85	42.82	11.64
Employed (0–1)	.87	–	.95	–	.97	–
Pre-parenthood work hours (0–80)	–	–	41.49^a^	10.29	41.94^a^	11.46
Transition to parenthood (0–1)	0	–	0	–	1	–
Pregnancy (0–1)	0	–	.10	–	0	–
Months since birth/12 (0–11)	0	.00	0	.00	3.38	2.54
Age (18–51)	30.66	7.65	31.26	5.04	36.28	5.13
Single (0–1)	.66	–	.28	–	.02	–
Cohabiting (0–1)	.20	–	.37	–	.10	–
Married (0–1)	.14	–	.35	–	.88	–
Self-rated health (1–5)	4.23^a^	.62	4.32	.58	4.20^a^	.59
*N* observations	5098		1005		1016	
*N* individuals	1038		294			

^a^Means do not differ across groups (two-tailed mean comparison *t* tests, *p* < .05)

**Table 2:** Well-being, parenthood variables, lifestyle variables, and parental background variables: descriptive statistics for women

Variable (range)	No transition to parenthood		Pre-Parenthood		After Parenthood	
	Mean	SD	Mean	SD	Mean	SD
Well-being (0–10)	7.70	2.03	8.07^a^	1.83	7.97^a^	1.82
Optimism (0–10)	7.28^a^	1.59	7.58	1.48	7.32^a^	1.48
Life satisfaction (0–10)	7.89	1.38	8.32	1.23	8.15	1.28
Satisfaction with free time (0–10)	6.73^a^	2.40	6.71^a^	2.24	5.98	2.61
Satisfaction with leisure act (0–10)	7.66^a^	1.97	7.80^a^	1.91	6.79	2.34
Leisure (0.00–3.18)	1.91	.39	1.97	.37	1.80	.42
Pre-parenthood leisure (0.82–3.00)	–	–	1.98	.31	1.97	.34
Work hours (0–85)	33.14	15.62	35.19^a^	14.55	17.14^a^	14.44
Employed (0–1)	.89	–	.91	–	.77	–
Pre-parenthood work hours (0–80)	–	–	35.19^a^	11.46	35.75^a^	12.61
Transition to parenthood (0–1)	0	–	0	–	1	–
Pregnancy (0–1)	0	–	.12	–	0	–
Months since birth/12 (0–11.08)	0	.00	0	.00	3.32	2.51
Age (18–51)	30.90	7.70	28.92	4.50	34.35	4.81
Single (0–1)	.60	–	.24	–	.03	–
Cohabiting (0–1)	.24	–	.40	–	.09	–
Married (0–1)	.16	–	.36	–	.88	–
Self-rated health (1–5)	4.14^a^	.66	4.27	.63	4.16^a^	.61
*N* observations	4638		997		1190	
*N* individuals	942		330			

^a^Means do not differ across groups (two-tailed mean comparison *t* tests, *p* < .05)

Within the observation window of this study about a quarter of the people makes the transition to parenthood (294 men, 22.1 % and 330 women, 25.9 %). Among both men and women, the group who does not make the transition to parenthood shows the lowest score on well-being. Parents report higher levels of well-being before the transition to parenthood, but their well-being score decreases after the transition. On average, women report lower levels of well-being. Thus, we find support for the idea that the transition into parenthood has a negative effect on well-being, although parents remain happier than the childless comparison group. Both parents who did not yet make the transition and those who remain childless report similar levels of leisure. After becoming a parent, involvement in leisure goes down as expected. Regarding work hours, different patterns emerge for men and women. Both future mothers and fathers work more hours before the transition compared to their childless counterparts. Mothers reduce their work hours after the transition from about 35 to about 17-h a week, whereas fathers remain working about the same number of hours (42-h a week).

The average age for men who do not become parents is slightly lower compared to the parents (30.7 years compared to 31.3 years before the transition). For women, the opposite applies (31 vs. 29 years). The patterns in partner status are predictable: In the majority of observations of individuals who do not become parents, the respondent is single (66 % for men and 60 % for women). Non-married cohabitation is most common among parents before the transition (37 % for men, 40 % for women), whereas in the majority of observations of parents, the respondents are married (88 % for men and women). Ten percent of the observations of men and twelve percent of the observations of women pertain to the period in which the woman was pregnant. The average time since the birth is about 3.3 years for both men and women.

Not presented in the table are differences in background characteristics such as education and occupational status. Compared to the childless group, the group who will become parents tends to be higher educated (both men and women) and has a higher occupational status (men only). These characteristics are not included in the fixed effect models because they mainly vary across individuals and are fairly constant over time.

### Explanatory Analyses

**Fig. 1:**
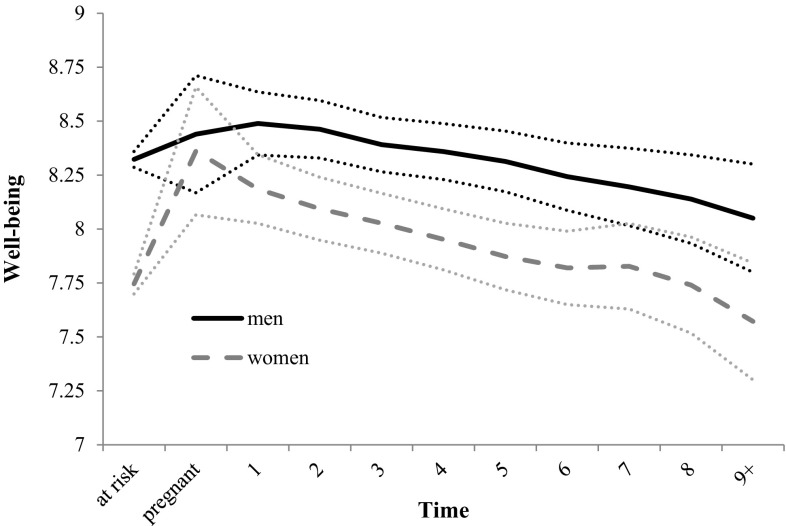
Predicted well-being and after having had a child (95 % confidence intervals by *dotted lines*). *X*-axis is defined as at risk of birth, (partner) pregnant, and years since birth. Based on Model 1

The results of the explanatory analyses are depicted in Table 3 (for men) and Table 4 (for women). Model 1 is the baseline model and investigates the relationship between becoming a parent and well-being by including a set of variables that captures this life course transition. Figure 1 shows the predicted effect of the transition into parenthood (on the original unstandardized scale) for men and women separately based on Model 1. As is clear from this figure, men’s well-being was not affected by becoming a father, nor is it associated with the pregnancy of their partner or the time since becoming a father. For women, we find that well-being increased when they were pregnant, by about a quarter standard deviation (.237, which translates into about ½ point on the original scale). It also increased once they entered motherhood but to a lesser extent (.161). The negative effect of years since birth (−.021) indicates that the beneficial effect of having a child is attenuated over time: In about eight years, the point-estimate of the net impact of motherhood on distress is zero (.161/.021 ≈ 8 years). Women’s well-being only approaches that of men around the time of birth. Figure 1 also demonstrates that after about five years, mothers are no longer significantly happier than women still at risk of motherhood. Summarizing, Hypotheses 1a and 1b are both rejected for men and Hypothesis 1b that predicted an increase in well-being is confirmed for women.

**Table 3:** Fixed effects model results predicting well-being for men (*N* = 7119 observations from 1332 individuals)

Predictors	Model 1		Model 2		Model 3		Model 4		Model 5	
	*B*	SE	*B*	SE	*B*	SE	*B*	SE	*B*	SE
*Controls*										
Age^a^	−.009*	.004	−.009*	.004	−.009*	.004	−.009*	.004	−.010**	.004
Single	−.263***	.047	−.270***	.047	−.270***	.047	−.270***	.047	−.267***	.047
Cohabiting	−.135**	.043	−.143***	.043	−.144***	.043	−.143***	.043	−.143***	.043
Married (ref.)	–		–		–		–		–	
Self-reported health^a^	.151***	.017	.150***	.017	.151***	.017	.150***	.017	.149***	.017
*Parenthood characteristics*										
Pregnancy of partner	−.100	.076	−.095	.076	−.096	.076	−.096	.076	−.094	.076
Parenthood	−.055	.049	−.055	.049	−.045	.049	−.045	.049	−.038	.049
Months since birth/12	−.013	.010	−.013	.010	−.013	.010	−.013	.010	−.012	.010
*Pre*-*birth lifestyle* *×* *parenthood interactions*										
Leisure^a^			−.086*	.038	−.085*	.038	−.089*	.041	−.082*	.041
Work hours^a^					−.038	.037	−.039	.038	−.026	.038
Leisure^a^ × work hours^a^							.011	.043	.008	.043
*Time*-*varying lifestyle characteristics*										
Leisure^a^									.021	.013
Work hours^a^									.048*	.020
Employed									−.026	.056
Constant	−.048	.149	−.035	.149	−.034	.149	−.034	.149	.017	.156

* *p* < 0.05; ** *p* < 0.01; *** *p* < 0.001

^a^Standardized variable

**Table 4:** Fixed effects model results predicting well-being for women (*N* = 6825 observations from 1272 individuals)

Predictors	Model 1		Model 2		Model 3		Model 4		Model 5	
	*B*	SE	*B*	SE	*B*	SE	*B*	SE	*B*	SE
*Controls*										
Age^a^	−.005	.004	−.005	.004	−.005	.004	−.005	.004	−.005	.004
Single	−.089	.054	−.092	.054	−.094	.054	−.099	.054	−.100	.054
Cohabiting	−.021	.049	−.023	.049	−.028	.050	−.035	.050	−.038	.050
Married (ref.)	–		–		–		–		–	
Self-reported health^a^	.224***	.018	.224***	.018	.224***	.018	.224***	.018	.224***	.018
*Parenthood characteristics*										
Pregnancy	.237**	.083	.242**	.083	.239**	.083	.228**	.083	.229**	.083
Parenthood	.161**	.054	.161**	.054	.151**	.055	.144**	.055	.168**	.059
Months since birth/12	−.021*	.011	−.021*	.011	−.021	.011	−.021	.011	−.020	.011
*Pre*-*birth lifestyle* *×* *parenthood interactions*										
Leisure^a^			−.029	.035	−.029	.035	−.053	.036	−.051	.036
Work hours^a^					−.030	.037	−.032	.037	−.024	.038
Leisure^a^ × work hours^a^							−.085**	.032	−.086**	.032
*Time*-*varying lifestyle characteristics*										
Leisure^a^									.006	.016
Work hours^a^									.021	.022
Employed									−.013	.052
Constant	−.896***	.168	−.896***	.168	−.890***	.168	−.888***	.168	−.863***	.176

* *p* < .05; ** *p* < .01; *** *p* < 0.001

^a^Standardized variable

Model 2 investigates whether the impact of parenthood on well-being varies with pre-parenthood leisure frequency. For men (Table 3), the negative interaction shows that men with more active lifestyles experience a decrease in well-being after becoming a father. The effect size of −.086 is relatively small (all variables were standardized), but this finding supports Hypothesis 2a. For women (Table 4), well-being does not appear to be contingent upon leisure before parenthood.

In Model 3, we also included an interaction between parenthood and pre-parenthood work hours. Hypothesis 2b (predicting a moderating effect of pre-parenthood work hours) is rejected for both men and women. For men, the pre-parenthood leisure interaction remains significant, which suggests that the moderating effect for men cannot be accounted for heterogeneity in their paid work involvement. For women, including this interaction did not alter the results either. Note that an additional model that only included the interaction with work (and excluded the interaction with leisure) did not show a moderating effect for work (results not reported).

In Model 4, we take a closer look at the role of leisure and work before parenthood by including a three-way interaction between parenthood, pre-parenthood leisure, and pre-parenthood paid work and testing Hypothesis 3. For men, this interaction is not significantly different from zero. For women, there is a significant negative interaction effect. This negative interaction term (−.085) suggests that the beneficial effect of becoming a mother on well-being (.144) is weaker for women who combined relatively high levels of leisure with relatively long work hours before becoming a mother. Thus, Hypothesis 3 is confirmed for women, but only if the moderating effect of prior work hours is taken into account.

**Fig. 2:**
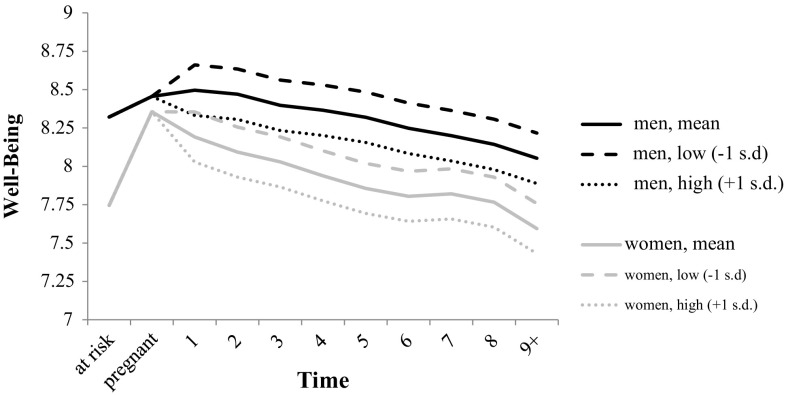
Interactions with pre-parenthood lifestyle: Predicted well-being by low (−1 SD), mean and high (+1 SD) pre-parenthood leisure for men (Model 2) and low (−1 SD), mean and high (+1 SD) combination of pre-parenthood leisure and work for women (Model 4). *X*-axis is defined as at risk of birth, (partner) pregnant and years since birth

The main findings with regard to Hypothesis 2 are depicted in Fig. 2. This figure shows the predicted well-being for men and women around the transition into parenthood by pre-parenthood lifestyle. We show the interaction with pre-parenthood leisure for men (Model 2) and the three-way interaction with pre-parenthood leisure and pre-parenthood work for women (Model 4). Figure 2 shows that the moderating effect of pre-parenthood lifestyles is similar for men and women. The most active men and women do not reap well-being benefits of becoming a parent. Instead, they experience a decline in well-being following parenthood. Note that for *women* this effect pertains to the three-way interaction of work, leisure, and parenthood, whereas for *men* this effect pertains to the two-way interaction with leisure. Thus, the effect of work hours seems to suppress the effect of leisure, but only for women. Women may be better able to cope with the time demands of a baby because they generally work fewer hours than men before parenthood, but this does not hold for women who work relatively long hours. Once, we consider this and take into account that all men worked relatively long hours (about 40 h on average vs. 35 for women, see Table 1 for details), the patterns are very similar for men and women who work long hours.

Model 5 extends Model 4 by including the time-varying leisure and work involvement measures. Men who increased their work hours experienced an increase in well-being, but the leisure interaction remains (the effect size changes slightly from −.089 to −.082), which suggests that this effect is not related to parenthood. For women, these variables were not related to well-being and the results remain the same. The results of this additional model suggest that it is the pre-parenthood lifestyle that matters (especially leisure involvement), regardless of changes in behavior following parenthood.

### Robustness

We ran a number of robustness checks (results not reported). First, we tested whether the gender differences were significantly different by pooling the models for men and women. The results suggest that the main parenthood effect and the moderating effects of pre-parenthood leisure and work indeed differed significantly. Second, we investigated whether the results would change if we conceptualized “work” as the combination of paid and unpaid work, which may be especially relevant for women. Results were very similar. Third, we obtained similar results if we took the average of only the three years before becoming a parent as pre-parenthood measures instead of averaging over all the years. Fourth, the dependent variable was quite skewed as relatively few responses scored less than 6 on the 0–10 scale (5 % had a 4 or less and 17 % a 6 or less). Results were very similar even if we collapsed the answer categories 0–4 or 0–6.

### Satisfaction with Life, Leisure, and Free Time

Finally, we ran additional analyses with alternative outcome measures to study whether the results apply to other indicators of well-being. Analyses for life satisfaction, satisfaction with leisure activities and satisfaction with free time (results available upon request) showed that results do not replicate for life satisfaction in general. Nevertheless, the results were quite similar for satisfaction with leisure and free time.

## Discussion

The current study aimed to increase our understanding of the association between parenthood and well-being by examining the role of lifestyles, and leisure participation in particular. Instead of assuming that parenthood is always difficult to reconcile with leisure and work, we argued that parents who participated less in leisure before the transition to parenthood would find it easier to adapt to parenthood. By acknowledging lifestyle differences between parents-to-be and theorizing on possible coping mechanisms, our study adds to the literature on parenthood and well-being.

Based on fixed effects models, using the Swiss Household Panel (*N* = 1332 men and 1272 women who were followed for 5.4 years on average), we can draw three main conclusions. First, we found that—on average—there was no relationship between parenthood and well-being for men, but for women, we found a positive effect that attenuated over time. This finding is in line with previous findings in Switzerland (Rizzi and Mikucka 2014). Second, these average effects appeared to hide considerable heterogeneity. Parenthood decreased well-being for men who more actively participated in leisure before the transition to parenthood and for women who, before the transition to parenthood, combined high levels of leisure involvement with a high level of labor force participation. Third, the lifestyle before parenthood was an important determinant of how childbirth affected well-being. Whereas prior lifestyle is important for how the transition to parenthood affects well-being, the level of change in lifestyle following parenthood does not seem to explain the observed patterns. Summarizing, we show that part of the heterogeneity in the effects of parenthood is related to parents’ lifestyle prior to parenthood.

Our study indicates that there is no best practice for active parents to deal with the transition to parenthood. Parenthood appears to be harmful to well-being for individuals with more active lifestyles, not because they are more or less likely to change their time use (as prior research suggests), but because their well-being decreases regardless of their strategies to deal with this transition. If these individuals scale back, they lose the benefits of an active lifestyle, but if they do not, the combination of demands and activities creates role overload. In contrast, individuals with less intensive leisure patterns or less demanding jobs seem to have more flexibility to accommodate the demands of children and, as a result, do not experience the same costs of parenthood.

A comparison of the results for men and women reveals a surprising similarity. It seems that both men and women who combined an active leisure pattern with long work hours experience more difficulties adapting to parenthood. This is in line with the literature showing that work-oriented women are less inclined to have children (Vitali et al. 2009). The similarity between men and women is interesting considering the fact that the work–family literature often stresses the differences. Yet, these analyses are usually restricted to the work–family interface and neglect time spent in leisure. Our study suggests that if we broaden the scope to consider leisure, we can detect gender similarities that we would otherwise miss. Women may experience difficulties combining parenthood with a career, but if they work fewer hours they do seem to be able to combine parenthood and leisure. Men, on the other hand, may be able to combine parenthood and a career, but seems to find it difficult to reconcile this with a strong involvement in leisure. These findings suggest fathers’ well-being would benefit from extended paternity leave as this would provide them with additional time to better balance parenthood, paid work, and leisure.

The results of this study are interesting in the light of prior research. First and foremost, it adds to the literature on the costs and benefits of parenthood (e.g., Nelson et al. 2014; Nomaguchi and Milkie 2003). The literature increasingly acknowledges there is substantial heterogeneity in the well-being effects of the transition to parenthood. Whereas prior research mostly considered socio-demographic factors, this study suggest that behavioral factors play a role as well. This study suggest that choices made earlier in life about how to spend time on leisure and work shape how later life transitions are felt and perceived, as these choices set a certain life style that becomes part of people’s expectations. Second, our findings are in line with earlier studies that showed that role overload can have detrimental consequences (e.g., Bianchi and Milkie 2010), but also suggests that scaling back can have unintended consequences. Third, we nuance the general finding in leisure studies that leisure has a positive effect on health and well-being (Han and Patterson 2007). Whereas having an active lifestyle in itself may be beneficial for well-being, we show that when life changing events such as becoming a parent present themselves, negative consequences for well-being may occur. Several studies have shown that the transition to parenthood is accompanied by a decrease in total leisure time, and in time spent in particular leisure activities, with typically stronger decreases in leisure time for women compared to men (Cantwell and Sanik 1993; Nomaguchi and Bianchi 2004; Dribe and Stanfors 2009). These studies often suggest that it is the scaling down that affects well-being, but our findings show that for well-being the lifestyle individuals have prior to becoming a parent is more defining than knowing how much leisure time they have lost in the process.

Although our findings are in line with the theoretical expectations, our conclusions should be considered with care. Analytically, we cannot explain the observed moderating factors. Are the detrimental effects of scaling back outweighed by the beneficial effects or is there another mechanism at play? Moreover, we cannot exclude the possibility that the anticipated costs and gains of becoming a parent affect both the likelihood of making the transition and pre-parenthood leisure, which might bias our results in a direction that is unclear. Even sophisticated fixed effects models do not protect against this (Kravdal 2014). Furthermore, we relied on a single-item measure of well-being, whereas multiple-item constructs are much to be preferred.

This study focused on the frequency of leisure, but it would be valuable if future research could explore the role of the nature of leisure. Because some activities are more compatible with children than others, not all types of leisure will decrease. Preliminary analyses (not shown) suggest that leisure close to the home (such as reading) does not decrease as much and that music and walking even show a slight increase. Thus, our conclusions may not apply to leisure in general, but to specific types of leisure. Future research could also investigate if and under what conditions the inclusion of children in leisure facilitates the transition to parenthood, and whether the mechanisms are different for fathers and mothers. Mothers may be more sensitive for changes in the nature of leisure because they are more likely than fathers to include children in their leisure activities and face more restrictions when pursuing individual independent leisure activities (Mattingly and Bianchi 2003).

Future research could also explore the role of the country context. Family and labor policies facilitate the combination of parenthood and paid work (Begall and Mills 2011; Vitali et al. 2009), and this may have indirect implications for parents’ leisure. In Switzerland, part-time work among women is relatively common (Ernst Stahli et al. 2009) and government support for families is limited and mostly oriented toward the traditional breadwinner family (Bonoli 2008). More elaborate, work–family policies may enhance parents’ quality of life because they help them to reconcile paid work and family demands, while at the same time enabling parents to remain involved in leisure. Cultural norms with regard to leisure may also play a role. Parenthood may be less restrictive in countries where it is more accepted to combine the care of children with leisure (e.g., bringing children to pubs and restaurants). It is also interesting to look at the couple level, since it is easier to maintain an active lifestyle if the partner scales back and takes up a substantial proportion of the child care. Finally, future research could study whether the effects are stronger for those who did not intend to have a child.

To conclude, this study both confirms and nuances prior research on parenthood and well-being. Whereas prior studies assumed that adapting one’s lifestyle to parenthood is one of the main costs of parenthood that all new parents have to face, our study showed that it is important to acknowledge that there is substantial variation. This finding is in line with the general notion that parents are a heterogeneous group whose specific circumstances moderate the impact of parenthood on well-being. Nevertheless, whereas prior research centered on variation along socio-demographic characteristics, such as age and marital status, our study shows that it is crucial to consider lifestyle in order to come closer to an understanding of heterogeneity in the parenthood effect.
